# HIV-1 Tat Regulates Occludin and A*β* Transfer Receptor Expression in Brain Endothelial Cells via Rho/ROCK Signaling Pathway

**DOI:** 10.1155/2016/4196572

**Published:** 2016-08-02

**Authors:** Yanlan Chen, Wen Huang, Wenlin Jiang, Xianghong Wu, Biao Ye, Xiaoting Zhou

**Affiliations:** ^1^Department of Neurology, First Affiliated Hospital, Guangxi Medical University, Nanning 530021, China; ^2^Department of Vasculocardiology, First Affiliated Hospital, Guangxi Medical University, Nanning 530021, China

## Abstract

HIV-1 transactivator protein (Tat) has been shown to play an important role in HIV-associated neurocognitive disorders. The aim of the present study was to evaluate the relationship between occludin and amyloid-beta (A*β*) transfer receptors in human cerebral microvascular endothelial cells (hCMEC/D3) in the context of HIV-1-related pathology. The protein expressions of occludin, receptor for advanced glycation end products (RAGE), and low-density lipoprotein receptor-related protein 1 (LRP1) in hCMEC/D3 cells were examined using western blotting and immunofluorescent staining. The mRNA levels of occludin, RAGE, and LRP1 were measured using quantitative real-time polymerase chain reaction. HIV-1 Tat at 1 *µ*g/mL and the Rho inhibitor hydroxyfasudil (HF) at 30 *µ*mol/L, with 24 h exposure, had no significant effect on hCMEC/D3 cell viability. Treatment with HIV-1 Tat protein decreased mRNA and protein levels of occludin and LRP1 and upregulated the expression of RAGE; however, these effects were attenuated by HF. These data suggest that the Rho/ROCK signaling pathway is involved in HIV-1 Tat-mediated changes in occludin, RAGE, and LRP1 in hCMEC/D3 cells. HF may have a beneficial influence by protecting the integrity of the blood-brain barrier and the expression of A*β* transfer receptors.

## 1. Introduction

Tight junction (TJ) proteins are essential components of blood-brain barrier (BBB) integrity [1, 2]. Signaling between pericytes and endothelial cells is critical for BBB maintenance [3, 4]. Occludin was the first integral membrane protein to be identified within TJs. Occludin has four transmembrane domains and a long COOH-terminal cytoplasmic domain (domain E). Occludin itself can localize at TJs and directly associates with ZO-1 [5]. Occludin plays a pivotal role in maintaining the homeostasis of the central nervous system [2], and its destruction increases the diffusion of viruses and other microorganisms across the BBB [6].

BBB dysfunction appears to be a particularly important component of HIV-associated neurocognitive disorders (HAND) [7]. HIV-1 transactivator protein (Tat) can be actively released from HIV-1-infected cells and easily cross cell membranes and the BBB, causing HAND [8, 9]. High levels of extracellular HIV-Tat have been recently reported in cerebrospinal fluid of HIV-infected patients [10]. HIV-1 Tat has been shown to disrupt TJs associated with astrocytes along the BBB, increasing the permeability of the barrier [11]. HIV-1 exposure also increases intracellular levels of amyloid-beta (A*β*) in human cerebral microvascular endothelial cells (hCMEC/D3) [12]. The BBB plays a critical role in both HIV-1 and A*β* pathology [13]. BBB disruption mediates some of the tissue damage that accompanies HIV-1 infection of the brain and so facilitates entry of the virus into the central nervous system [14]. The BBB endothelial cells respond to inflammatory stimuli, such as cytokines and A*β*, ultimately causing BBB disruption. Furthermore, the BBB plays a multifaceted role both upstream and downstream of the amyloid cascade to cause inflammation and oxidative stress, which can promote the accumulation of A*β* in the brain [15]. Several HIV-1 proteins have been shown to be amyloidogenic. HIV-1 Tat protein has been reported to inhibit A*β*-degrading enzyme neprilysin, leading to increased levels of soluble A*β* in cell culture [16].

Ras homolog gene family, member A (RhoA), is a small guanosine triphosphate-binding protein. Rho-associated kinase (ROCK) was the first downstream effector of Rho to be identified [17], and ROCK has been reported to mediate BBB disruption [18]. Rho-ROCK signaling was involved in mural cell recruitment to the vessel wall in brain, which was involved in maintaining BBB integrity [19]. Hydroxyfasudil (HF), a specific inhibitor of ROCK with strong effectiveness and selectivity [20, 21], promotes neuronal regeneration and is clinically used in patients with disorders such as spinal-cord injuries and stroke [22, 23]. Therefore, ROCK can be considered a promising molecular target for the treatment of neurological diseases [24, 25].

However, whether the Rho/ROCK signaling pathway is involved in HIV-induced BBB disruption and the expression of A*β* transfer receptors is not completely understood. Therefore, the aim of the present study was to evaluate the relationship between occludin, A*β* transfer receptors, and the Rho/ROCK signaling pathway in hCMEC/D3 cells.

## 2. Materials and Methods

### 2.1. HIV-1 Tat

Recombinant HIV-1 Tat clade B protein (Prospec, Rehovot, Israel), produced in* Escherichia coli*, is formed of a single, nonglycosylated, polypeptide chain containing 86 amino acids encoded by exons, with a molecular mass of 14 kDa. The amino acid sequence is as follows: MEPVDPRLEP WKHPGSQPKT ACTNCYCKKC CFHCQVCFIT KALGISYGRK KRRQRRRPPQ GSQTHQVSLS KQPTSQSRGD PTGPKE. It is recommended that lyophilized HIV-1 Tat is stored desiccated below −18°C and reconstituted in sterile 18 MΩ-cm H_2_O.

### 2.2. Cell Cultures

hCMEC/D3 cells were provided as a gift from Dr. P.-O. Couraud (Institut Cochin, Paris, France). Endothelial basal medium (EBM)-2 (Lonza, Walkersville, MD, USA) was supplemented with 5% fetal bovine serum (Lonza, Switzerland), 1 ng/mL basic fibroblast growth factor (Sigma-Aldrich, USA), 5 *µ*g/mL ascorbic acid (Sigma-Aldrich), 1/100 chemically defined lipid concentrate (Gibco, NY, USA), 10 mmol/L HEPES (Beyotime, Jiangsu, China), 1.4 *µ*mol/L hydrocortisone (Sigma-Aldrich, USA), and 1% penicillin-streptomycin (Beyotime), as recommended by the manufacturer (Lonza, Walkersville, MD, USA). This solution is called complete EBM-2 medium. hCMEC/D3 cells were seeded into flasks coated with 1 mg/mL collagen type I (R&D Systems) and cultured in complete EBM-2 medium, maintained at 37°C in a humidified atmosphere of 5% CO_2_.

### 2.3. Cell Viability

hCMEC/D3 cells at the exponential growth phase were seeded onto 96-well plates at a density of 1 × 10^4^ cells/well in 200 *μ*L serum-free medium and treated with HIV-1 Tat at various concentrations (0, 0.25, 0.5, 1, or 1.250 *µ*g/mL), heat-inactivated Tat, or the Rho inhibitor HF (0, 10, 30, 50, 80, or 100 *μ*mol/L; Tianjin Chase Sun Pharmaceutical Co, Tianjin, China) for different periods of time (0, 6, 12, 24, or 30 h). The cells were incubated with 15 *µ*L 3-(4,5-dimethylthiazol-2-yl)-2, 5-diphenyltetrazolium bromide solution (MTT, 5 mg/mL; Sigma-Aldrich, USA) for additional 4 h at 37°C and 5% CO_2_. The optical density in each well was measured at 570 nm using a 96-well plate reader (Thermo Scientific, USA). HIV-1 Tat at 1 *µ*g/mL and HF at 30 *μ*mol/L for 24 h had no effect on the viability of hCMEC/D3 cells, so these concentrations were used in subsequent experiments.

hCMEC/D3 cells were pretreated with 30 *μ*mol/L HF for 2 h prior to exposure to 1 *µ*g/mL HIV-1 Tat, and cells were then incubated for 24 h without serum. The HIV-1 Tat concentrations used in the present study are consistent with data from the literature, which indicate that concentrations of Tat in HIV-infected patients can reach the range of *µ*g/mL of serum [26]. Heat-inactivated Tat (as another control) was obtained by heating the protein at 90°C for 1 h, which inactivates the biological potentials of Tat.

### 2.4. Western Blot Analysis

Treated endothelial cultures were washed three times and lysed in a RIPA cell lysis buffer (Beyotime) containing protease inhibitor cocktail tablets (Beyotime). The lysates were centrifuged at 12,000 ×g for 15 min, the supernatants were collected, and protein concentrations were measured using the BCA Protein Assay Kit (Beyotime). Total proteins were mixed with 5x SEMS-PAGE protein sample buffer solution (Beyotime), then boiled for 5 min at 100°C, and stored at −20°C until use. Equal masses of proteins (20 *μ*g) were separated on SDS-polyacrylamide gel and electrophoresed. Proteins were blotted onto polyvinylidene fluoride membranes (0.22/0.45 *μ*m; Millipore, Billerica, MA, USA). The membranes were blocked with 5% fat-free milk at room temperature for 1 h and then incubated at 4°C overnight with different primary antibodies diluted in primary antibody dilution buffer (Beyotime). The primary antibodies were as follows: occludin (1 : 1000, mouse monoclonal antibody; Invitrogen, Carlsbad, CA, USA), RAGE (1 : 1000, rabbit monoclonal antibody; Abcam), LRP1 (1 : 10,000, rabbit monoclonal antibody; Abcam), and GAPDH (1 : 10,000; Proteintech Group, Chicago, IL, USA). The membranes were then incubated for 1 h with IRDye 680RD goat anti-rabbit immunoglobulin (Ig) G and IRDye 680RD goat anti-mouse IgG secondary antibodies (both 1 : 10,000; LI-COR Biosciences, Lincoln, NE, USA) diluted in secondary antibody dilution buffer (Beyotime). Proteins were visualized by scanning the membrane on an Odyssey Infrared Imaging System (LI-COR Biosciences) with the 700 and 800 nm channels. The density of bands was calculated using Image–J software (National Institutes of Health, Bethesda, MD, USA). Protein levels were represented by the ratios of optical densities in their bands, normalized against GAPDH.

### 2.5. Reverse Transcription- (RT-) PCR and Quantitative Real-Time PCR

Total RNA was extracted from hCMEC/D3 cells using TRIzol reagent (Invitrogen) and reverse transcribed into cDNA using the Prime-Script RT reagent kit (Takara Bio, Dalian, Japan) according to the manufacturer's instructions. The resulting cDNA was used as a template for RT-PCR. The primer sequences were as follows: occludin (Invitrogen): 5′-TCAGGGAATATCCACCTATCACTTCAG-3′ and 5′-CATCAGCAGCAGCCATGTACTCTTCAC-3′; RAGE (Takara): 5′-CAACGGCTCCCTCTTCCTT-3′ and 5′-TTGGTCTCCTTTCCATTCCTGT-3′; LRP1 (Takara): 5′-CGCCTCCTACCACTTCCAAC-3′ and 5′-CGCCACCTCAATCACATCTC-3′; and GAPDH (Invitrogen): 5′-GCACCGTCAAGGCTGAGAAC-3′ and 5′-TGGTGAAGACGCCAGTGGA-3′. Quantitative RT-PCR was performed using a Taq PCR Master Mix Kit (Takara) and conducted on the ABI Prism 7500 sequence detection system (Applied Biosystems, USA) using RT Reaction Mix in a total volume of 20 *μ*L at 25°C for 10 min, 42°C for 30 min, and 94°C for 5 min. GAPDH served as an internal control in relative RT-PCR. The relative levels of the target genes were quantified using 2^−ΔΔCt^ method.

### 2.6. Immunofluorescence Microscopy

hCMEC/D3 cells were seeded onto circular glass coverslips in a 24-well plate and incubated for 24 h. The cells were fixed for 30 min with 4% paraformaldehyde (Solarbio, Beijing, China) on ice and permeabilized with 0.1% Triton X-100 (Beyotime) for 5 min. Samples were incubated in blocking solution of 3% bovine serum albumin (Sigma, USA) for 1 h at room temperature and in antibodies against LRP1 (1 : 200, rabbit monoclonal antibody; Abcam) and RAGE (1 : 200, rabbit polyclonal antibody; Abcam) at 4°C overnight. The secondary antibodies, Alexa Fluor-488 donkey anti-rabbit (1 : 200; Invitrogen) and Alexa Fluor 594 donkey anti-mouse (1 : 200; Invitrogen), were added and incubated for 2 h, then stained with 4′,6-diamidino-2-phenylindole (Invitrogen), and visualized using a fluorescence microscope (Nikon A1; Nikon).

### 2.7. Statistical Analysis

The results are expressed as means ± standard deviation. SPSS version 17.0 (SPSS, Chicago, IL, USA) was used to perform statistical analyses. Comparisons between groups were conducted using a parametric test (one-way analysis of variance) combined with a multiple comparison test (least-squares difference or the Bonferroni* post hoc *test). Statistical significance was set at *p* < 0.05.

## 3. Results

### 3.1. Cell Viability

**Figure 1:**
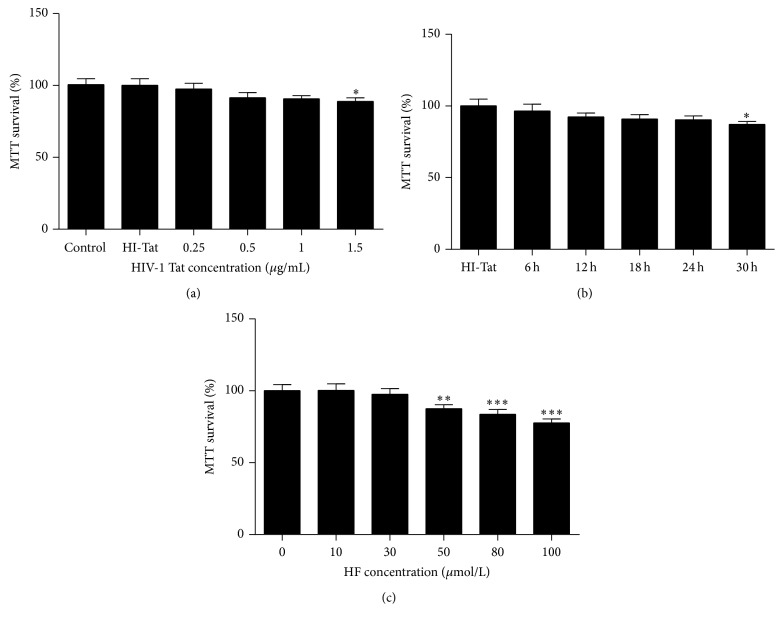
Cell viability. hCMEC/D3 cells were treated with HIV-1 Tat ((a), (b)) at different concentrations (0, 0.25, 0.5, 1, or 1.25 *µ*g/mL) or heat-inactivated Tat or HF (0, 10, 30, 50, 80, or 100 *μ*mol/L) (c) for different durations (0, 6, 12, 24, and 30 h), with cell viability detected using an MTT assay. Cell viability was not affected by 1 *µ*g/mL HIV-1 Tat or 30 *µ*mol/L HF for 24 h. Results are shown as means ± standard error of the mean (*n* = 5). ^*∗*^ *p* < 0.05, ^*∗∗*^ *p* < 0.01, and ^*∗∗∗*^ *p* < 0.001 versus control.

hCMEC/D3 viability was tested using an MTT assay. HIV-1 Tat at 1 *µ*g/mL and HF at 30 *µ*mol/L with 24 h exposure had no significant effect on hCMEC/D3 cell viability (Figure 1).

### 3.2. The RhoA/ROCK Signaling Pathway Is Involved in HIV-1 Tat-Induced Changes in Occludin Expression

**Figure 2:**
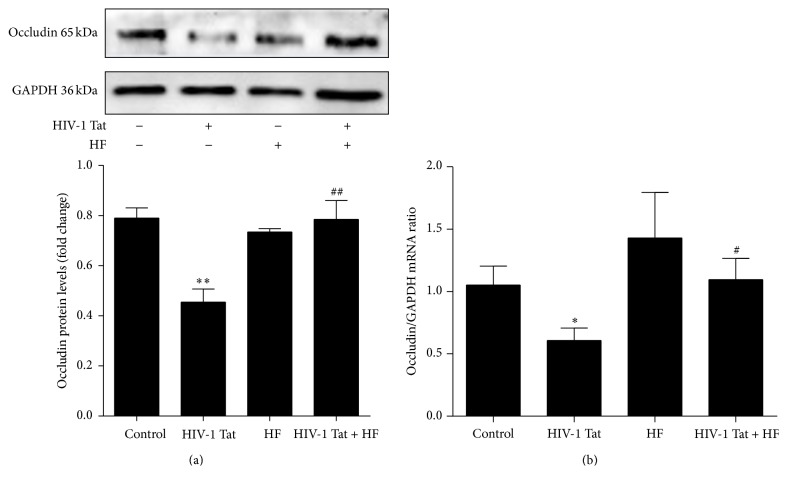
Role of RhoA/ROCK signaling in HIV-1 Tat-induced changes in occludin. hCMEC/D3 cells were pretreated with HF (30 *μ*mol/L) 2 h prior to the addition of occludin. HIV-1 Tat treatment was continued for 24 h for western blotting (a) and for 12 h for RT-PCR (b). HIV-1 Tat exposure was associated with decreased protein and mRNA levels of occludin in hCMEC/D3 cells. With coexposure to HF and HIV-1 Tat, occludin protein and mRNA levels were significantly increased when compared with exposure to HIV-1 Tat only. Data are expressed as means ± standard error of the mean (*n* = 3, values determined by the ratio to GAPDH, for (a), *n* = 5 for (b)). ^*∗*^ *p* < 0.05 and ^*∗∗*^ *p* < 0.01 versus control; ^#^ *p* < 0.05 and ^##^ *p* < 0.01 versus HIV-1 Tat.

To observe the effects of HIV-1 Tat on expression levels of the TJ protein occludin, hCMEC/D3 cells were exposed to 1 *µ*g/mL HIV-1 Tat for 24 h. The protein levels of occludin were significantly lower in the HIV-1 Tat group than in the control group (Figure 2(a)). With exposure to HIV-1 Tat for 12 h, the mRNA expression of occludin, as examined by real-time RT-PCR, was consistent with the protein levels (Figure 2(b)).

To explore whether RhoA/ROCK signaling is involved in HIV-1 Tat-induced downregulation of occludin, hCMEC/D3 cells were pretreated for 2 h with 30 *μ*mol/L HF, followed by coexposure to HF and 1 *µ*g/mL HIV-1 Tat for 24 h (for western blotting) or 12 h (for real-time RT-PCR). Occludin protein and mRNA levels were significantly increased with coexposure to HF and HIV-1 Tat compared with HIV-1 Tat only (Figures 2(a) and 2(b)).

### 3.3. HIV-1 Tat-Induced Changes in LRP1 and RAGE Expression

**Figure 3:**
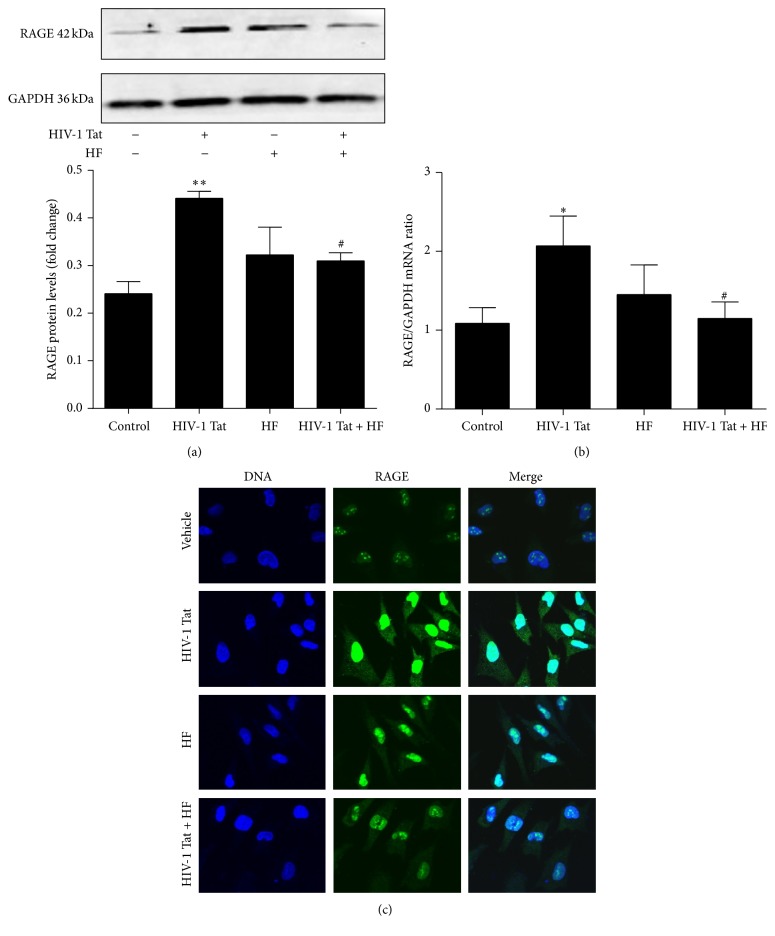
Role of RhoA/ROCK signaling in HIV-1 Tat-induced changes in RAGE. Exposure to HIV-1 Tat resulted in markedly higher levels of RAGE protein (a) and mRNA (b) and stronger immunoreactivity (c) compared with the untreated group. hCMEC/D3 cells were pretreated with HF (30 *μ*mol/L) 2 h prior to HIV-1 Tat treatment for 24 h. RAGE protein and mRNA levels and immunoreactivity were significantly decreased with coexposure to HF and HIV-1 Tat compared with HIV-1 Tat only. Data are expressed as means ± standard error of the mean (*n* = 3, values determined by the ratio to GAPDH, for (a), *n* = 5 for (b)). ^*∗*^ *p* < 0.05 and ^*∗∗*^ *p* < 0.01 versus control; ^#^ *p* < 0.05 versus HIV-1 Tat.

**Figure 4:**
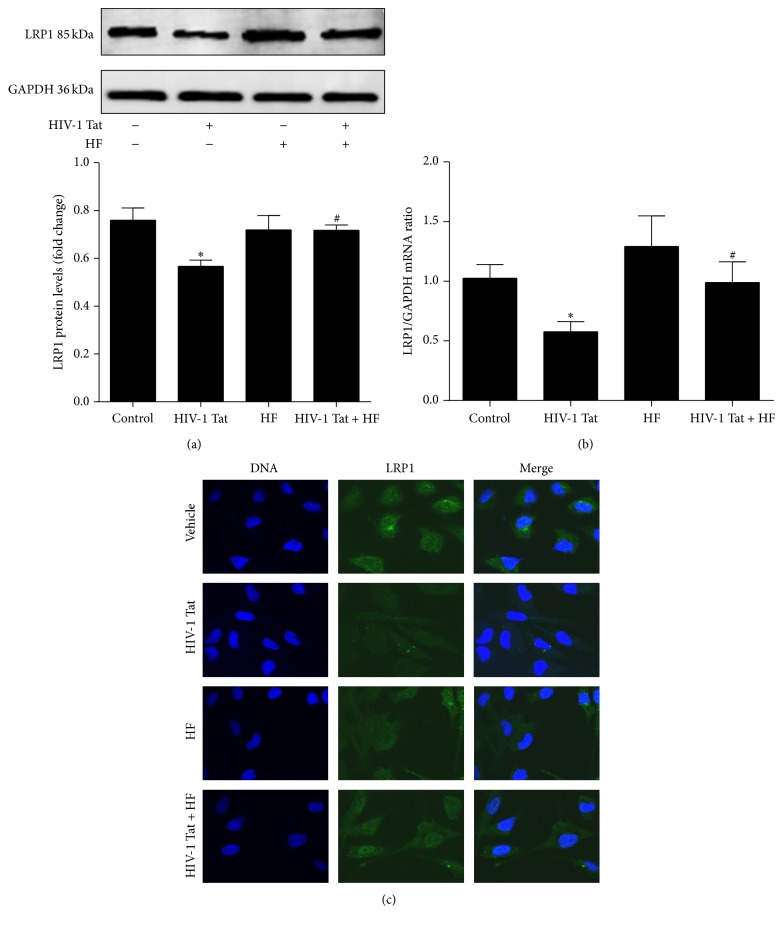
Role of RhoA/ROCK signaling in HIV-1 Tat-induced changes in LRP1. Exposure to HIV-1 Tat resulted in markedly lower levels of LRP1 protein (a) and mRNA (b) and weaker immunoreactivity (c) compared with the untreated group. hCMEC/D3 cells were pretreated with HF (30 *μ*mol/L) 2 h prior to HIV-1 Tat treatment for 24 h. LRP1 protein and mRNA levels and immunoreactivity were significantly increased with coexposure to HF and HIV-1 Tat compared with HIV-1 Tat only. Data are expressed as means ± standard error of the mean (*n* = 3, values determined by the ratio to GAPDH, for (a), *n* = 5 for (b)). ^*∗*^ *p* < 0.05 versus control; ^#^ *p* < 0.05 versus HIV-1 Tat.

To observe whether HIV-1 Tat could affect the expression of A*β* transporters in hCMEC/D3 cells, the expression of LRP1 and RAGE was examined using western blotting, real-time RT-PCR, and immunoreactivity. Western blotting showed that the expression of RAGE was significantly increased compared to the control group following treatment with 1 *µ*g/mL HIV-1 Tat, while LRP1 expression was downregulated (Figures 3(a) and 4(a)). Treatment with HIV-1 Tat also significantly decreased LRP1 mRNA and increased RAGE mRNA levels, consistent with the protein levels (Figures 3(b) and 4(b)), and resulted in markedly stronger RAGE and weaker LRP1 immunoreactivity compared with the untreated group (Figures 3(c) and 4(c)).

### 3.4. The RhoA/ROCK Signaling Pathway Is Involved in the HIV-1 Tat-Induced Regulation of LRP1 and RAGE Expression

To investigate whether HF can protect against HIV-1 Tat-induced changes in LRP1 and RAGE expression, hCMEC/D3 cells were cotreated with HF and HIV-1 Tat for 24 h (for western blotting and immunofluorescence staining) or for 12 h (for real-time RT-PCR). As shown in Figures 3 and 4, HF downregulated RAGE expression and increased LRP1 protein levels. These results were consistent with those for RAGE and LRP1 mRNA levels, which significantly differed in the groups cotreated with HF and HIV-1 Tat versus HIV-1 Tat only.

## 4. Discussion

Although the mechanisms by which HIV invades the brain process are not fully understood, alterations of TJ protein expression can contribute, at least in part, to this phenomenon [27]. HIV-1 Tat is known to trigger oxidative stress-dependent apoptosis of neurons both* in vitro *and* in vivo *[9, 28]. Exposure to HIV-1 Tat has also been shown to disrupt the integrity of the BBB and result in endothelial hyperpermeability and increased transendothelial migration, as reported in our previous* in vitro *[1] and* in vivo *studies [28]. Tat clade B is more neuropathogenic, disrupts the integrity of the BBB to a greater extent than Tat clade C [29], and was therefore used in the current study. Our results demonstrate that HIV-1 Tat at 1 *µ*g/mL and the Rho inhibitor HF at 30 *µ*mol/L, with 24 h exposure, had no significant effect on hCMEC/D3 cell viability (Figure 1).

Previous reports have indicated that ROCK plays a role in ischemic stroke and edema formation [30]. Rho signaling is involved in the regulation of TJs; Rho directly phosphorylates occludin and other TJ proteins [31]; and Rho-kinase inhibitor improves cerebral integrity and function by regulating endothelial cell oxidative stress and reorganizing intercellular junctions after acute ischemic attacks [30]. In addition, inhibition of Rho activity has been reported to protect against Tat-induced alterations in total and nuclear ZO-1 protein levels [32], while ROCK inhibitor has been reported to preserve occludin and ZO-1 levels in the brain or primary human brain microvascular endothelial cells [33, 34]. In the present study, HF upregulated the protein and mRNA levels of occludin, the destruction of which was induced by HIV-1 Tat (Figures 2(a) and 2(b)). HF protected against HIV-1 Tat-mediated BBB dysfunction partly by inhibiting the RhoA/ROCK signaling pathway.

HIV-Tat is a ligand for LRP1 and it may compete with A*β* leading to decreased clearance of A*β* from the brain and promoting its deposition [35]. HIV-1 Tat has also been reported to significantly increase A*β* levels in postmortem brain samples from patients infected with HIV-1 [16]. HIV-1 exposure increases the intracellular levels of A*β* in hCMEC/D3 cells [12]. HIV-1 Tat inhibits the uptake of A*β* by primary mouse microglial cells [36]. Furthermore, the induction of HIV-1 Tat in astrocytes has been shown to increase neuronal damage, tau phosphorylation, and A*β* plaque formation in APP/presenilin-1 transgenic mice [37], suggesting an important role for HIV-1 Tat in the development of HAND. However, the molecular mechanisms involved in HIV-1 Tat-evoked A*β* deposition in the brain remain largely unknown. Neurodegeneration and dementia in Alzheimer's disease may involve similar molecular mechanisms as those involved in HIV-associated dementia [12, 38].

**Figure 5:**
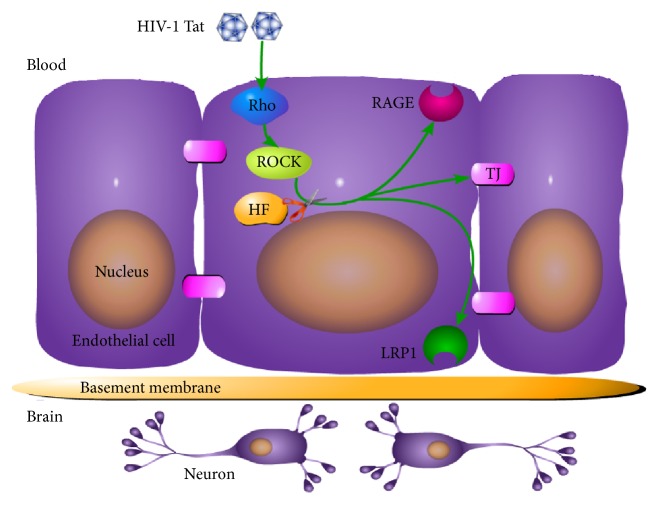
Schematic diagram illustrating the effects of HIV-1 Tat on occludin, RAGE, and LRP1 in brain endothelial cells. Exposure to HIV-1 Tat resulted in RAGE overexpression, which downregulated occludin and LRP1, stimulating the transfer of A*β* from the blood into the brain. HF protected against these effects. Exposure to HF inhibited HIV-1 Tat-induced dysregulation of occludin, RAGE, and LRP1.

However, the A*β* deposition pattern in HIV appears to be distinct from that of Alzheimer's disease, in which extracellular senile plaques are a predominant feature. In the HIV-infected brain, A*β* accumulates primarily as diffuse and intraneuronal deposits [39]. Peptides and proteins generally do not cross the BBB [40], but they can be transported into the brain via specific transport systems [41]. RAGE and LRP1 remain the most interesting targets. Transport of A*β* from the bloodstream into the brain is mediated by RAGE, while A*β* transport from the brain into the bloodstream is mediated by LRP1 [42, 43]. RAGE is a multiligand receptor in IgG superfamily that binds soluble A*β* and mediates pathophysiologically relevant cellular responses consequent to ligation by a variety of ligands [42]. LRP1 is a multiligand lipoprotein receptor that interacts with a broad range of secreted proteins and resident cell-surface molecules, mediating their endocytosis or activating signaling pathways through multiple cytosolic adaptor and scaffold proteins [44]. Increased RAGE and decreased LRP1 immunoreactivity within the microvasculature have been found in Alzheimer's disease patients [43]. Blocking RAGE with a specific neutralizing antibody can protect against the accumulation of exogenous A*β* in HIV-1-exposed hCMEC/D3 cells [12]. In the current study, 24 h exposure to HIV-1 Tat resulted in markedly stronger RAGE immunoreactivity and increased RAGE mRNA levels compared with the control group (Figure 3). This may directly lead to increased A*β* deposition in the brain. In contrast to the alterations in RAGE expression, exposure to HIV-1 Tat attenuated LRP1 levels in hCMEC/D3 cells (Figure 4). Our data suggest that HF may prevent the movement of A*β* into the brain and stimulate brain A*β* clearance across the BBB (Figure 5). The RhoA/ROCK signaling pathway plays a favorable role in maintaining A*β* homeostasis at the level of the BBB. However, different effects of HIV-1 on LRP1 have been observed. For example, HIV-1 p24 had no effects on LRP1 levels in an* in vitro* study [12]. The reason for this might be that different HIV subtype proteins affect LRP1 via multiple signaling pathways.

## 5. Conclusions

In the current study, the Rho-kinase inhibitor HF significantly inhibited HIV-1 Tat-induced occludin dysfunction and regulated LRP1 and RAGE expression in hCMEC/D3 cells, suggesting a potential protective role for HF in HIV-1 Tat-mediated BBB destruction and A*β* accumulation. A better understanding of the mechanisms involved in A*β* deposition in the brain during HIV-1 infection will help with the development of new therapeutic strategies for reducing the A*β* burden in HAND.
